# MRI of endometriosis in correlation with the #Enzian classification: applicability and structured report

**DOI:** 10.1186/s13244-023-01466-x

**Published:** 2023-07-05

**Authors:** Cristina Maciel, Hélder Ferreira, Dusan Djokovic, Jimmy Kyaw Tun, Jörg Keckstein, Stefania Rizzo, Lucia Manganaro

**Affiliations:** 1 Serviço de Radiologia, Centro Hospitalar Universitário São João, Porto, Portugal; 2 Departamento de Medicina, Faculdade de Medicina, Universidade do Porto, Alameda Prof. Hernâni Monteiro, 4200-319 Porto, Portugal; 3 Serviço de Ginecologia, Centro Materno Infantil do Norte, Centro Hospitalar Universitário do Porto, Largo do Prof. Abel Salazar, 4099-001 Porto, Portugal; 4 Instituto de Ciências Biomédicas Abel Salazar, Universidade do Porto, Rua de Jorge Viterbo Ferreira, 228, 4050-313 Porto, Portugal; 5 Maternidade Dr. Alfredo da Costa, Centro Hospitalar Universitário Lisboa Central (CHULC), Lisbon, Portugal; 6 Department of Obstetrics and Gynecology, NOVA Medical School – Faculdade de Ciências Médicas, NOVA University of Lisbon, Lisbon, Portugal; 7 Department of Obstetrics and Gynecology, Hospital CUF Descobertas, Lisbon, Portugal; 8 Department of Interventional Radiology, The Royal London Hospital, Barts Health NHS Trust, London, E1 1BB UK; 9 Scientific Endometriosis Foundation (Stiftung Endometrioseforschung/SEF), Westerstede, Germany; 10 Endometriosis Clinic Dres. Keckstein, Villach, Austria; 11 University of Ulm, Ulm, Germany; 12 Service of Radiology, Imaging Institute of Southern Switzerland, EOC, Via Tesserete 46, 6900 Lugano, Switzerland; 13 Faculty of Biomedical Sciences, Università Della Svizzera Italiana, Via G. Buffi 13, 6900 Lugano, Switzerland; 14 Department of Radiological, Oncological and Pathological Sciences, Sapienza University of Rome, Rome, Italy

**Keywords:** Endometriosis, #Enzian, Magnetic resonance imaging, Standardization, Structured report

## Abstract

Endometriosis represents one of the most common causes of life-impacting chronic pelvic pain and female infertility. Magnetic resonance imaging (MRI) plays an increasing role in the diagnosis and mapping of endometriosis, while diagnostic laparoscopy currently tends to be reserved for the patients with negative imaging results. The #Enzian, published in 2021, proposes a new comprehensive classification system of endometriosis, combining a complete staging of deep infiltrative endometriosis with the evaluation of peritoneal/ovarian/tubal localizations and the presence of adenomyosis. This article addresses in detail the applicability of the #Enzian classification, primarily based on surgical findings, to the MRI evaluation of the endometriosis. Overall, there is a significant matching between MRI features and the #Enzian classification criteria, two different perspectives of endometriosis mapping, with different goals and levels of detail. The main discrepancy lies in the evaluation of tubo-ovarian condition, which is not fully assessable by MRI. Furthermore, as endometriosis is a complex disease, usually multifocal, that can present with a myriad of imaging findings, MRI reporting should be clear and well organized. The authors group, both radiologists and gynecologists, propose a structured MRI report of endometriosis in correlation with the #Enzian classification, merging the detailed anatomical and pre-operative information provided by the MRI with the benefits of a comprehensive classification system of endometriosis in the clinical practice and research field.

**Critical relevance statement** This article addresses in detail the applicability of the #Enzian classification, primarily based on surgical findings, to the MRI evaluation of the endometriosis and proposes a #Enzian-based structured MRI report.

**Graphical abstract:**

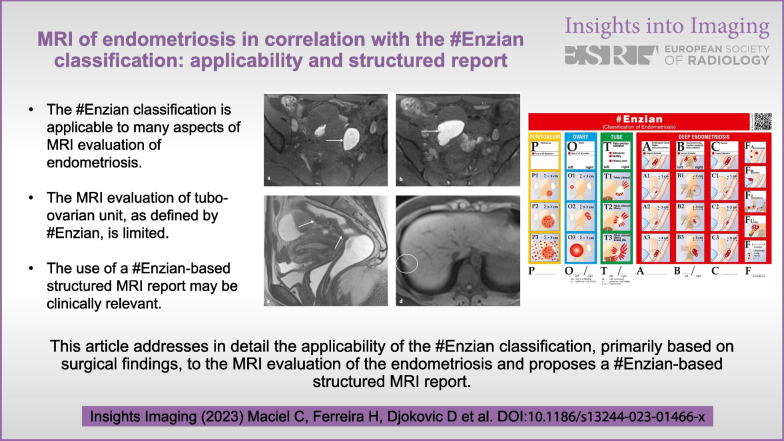

**Supplementary Information:**

The online version contains supplementary material available at 10.1186/s13244-023-01466-x.

## Key points

- The #Enzian is applicable to many aspects of MRI evaluation of endometriosis.
- The MRI evaluation of tubo-ovarian unit, as defined by #Enzian, is limited.
- The use of a #Enzian-based structured MRI report may be clinically relevant.

**Supplementary Information:**

The online version contains supplementary material available at 10.1186/s13244-023-01466-x.

## Background

Endometriosis, a gynecological disorder characterized by growth of endometrium-like tissue outside the uterine cavity, affecting around 10% of women of reproductive-age, is one of the most common causes of life-impacting chronic pelvic pain and female infertility [1]. Deep infiltrative endometriosis (DIE) is defined as deeper than 5 mm infiltration of tissue including the uterosacral ligaments, bowel or bladder [2].

Until recently, laparoscopic identification of endometriotic lesions with histological confirmation was recognized as the diagnostic gold standard [3], while ultrasound (US) and magnetic resonance imaging (MRI) were often used for pre-operative disease staging [4]. Due to availability and advances in the quality of imaging modalities, improved medical options, limited access to highly qualified surgeons as well as financial implications, diagnostic laparoscopy is currently recommended by the European Society for Human Reproduction and Embryology (ESHRE) only in patients with negative imaging results and/or when empirical treatment was inappropriate or unsuccessful [5].

In advanced centers, the sensitivity and specificity of both US and MRI (engaged by experienced operators) can exceed 80–90% for DIE [6–8]. Apart from the need to use a standardized imaging technique, we believe that the use of a clinically relevant, systematic and standardized approach for reporting imaging results is beneficial for radiologists, clinicians, researchers and patients. It may facilitate unambiguous communication between healthcare professionals, aid in patient counseling and management, and enable more appropriate and accurate data collection for scientific purposes [9]. The International Deep Endometriosis Analysis (IDEA) group published a consensus opinion in 2016, aiming to standardize the nomenclature and technique of US endometriosis evaluation [10]. In 2017, the European Society for Urogenital Radiology (ESUR) provided guidelines on patient preparation, MRI protocol and reporting criteria for the assessment of pelvic endometriosis [11].

Over the last five decades, numerous classifications of radiological and laparoscopic findings in women with endometriosis emerged [12–16]; however, broad international consensus has not yet been reached to indicate which of these systems should be recommended for routine use. The ideal classification system for endometriosis should describe the sites and extent of the disease, be related to surgical complexity and to disease-associated symptoms, including subfertility. Particularly important, it should be possible to use the same classification system both by imaging specialists and by surgeons [17].

To improve the description of DIE, the Enzian classification was developed in 2003 [18]. Acknowledging limitations in the original classification, this was subsequently revised into the “#Enzian” classification, which not only includes DIE, but also incorporates peritoneal lesions, ovarian endometriosis, and the extent of adnexal adhesions [14]. The #Enzian classification can be used pre-operatively (during the imaging studies) and intra-operatively, but also after surgical treatment for follow-up imaging assessment. Some endometriosis patients undergo more than one surgery [19], making a post-operative evaluation a possible pre-operative one. It can also be applied to patients conservatively managed, that undergo pharmacotherapy only, to monitor the treatment efficacy and clinical evolution.

The #Enzian classification and its previous versions have been recognized as a valid and suitable tool by a range of international and national societies [9]. While originally created for surgical classification, there is some evidence that it may be applicable to MRI reporting [20].

In this paper, we present the outcomes of a collaborative project between radiologists and surgeons to develop a standardized reporting system for MRI evaluation of endometriosis based on the #Enzian classification. The project involved three aspects: (1) to review of the existing practices and literature on standardized MRI reporting for endometriosis to identify any strengths and limitations (2) to evaluate the applicability of the #Enzian classification to MRI reporting and evaluation of endometriosis and (3) to develop a standardized reporting system drawing on the existing literature and expert consensus.

## Material and methods

### Literature review

A focused review of the existing literature regarding structured reporting in MRI for endometriosis and Enzian classification was performed. The search of available articles published in the English language up to August 2022 was performed using PubMed with the following criteria: (1) endometriosis AND magnetic resonance imaging AND structured reporting; and (2) endometriosis AND magnetic resonance imaging AND Enzian classification. All abstracts were analyzed, pertinent articles included and the references of all included articles were further reviewed in order to find additional relevant articles. A critical review of all included articles was performed with particular focus on methods used for producing structured reports (SR) and the incorporation of classification systems.

### Evaluating applicability of #Enzian criteria to MRI evaluation of endometriosis

Evaluation was performed based on expert consensus through an iterative process drawing on existing scientific literature on radiological-surgical correlation with respect to sensitivity and specificity of imaging.

### Generating standard MRI reporting system for endometriosis

The authors operated by consensus. Three radiologists, all with particular expertise in endometriosis, with 8 (C.M.), 16 (S.R.) and 22 (L.M.) years of experience in gynecological MRI, proposed a preliminary draft of a SR for MRI of endometriosis incorporating the #Enzian classification. This draft was reviewed by three gynecologists, (D.D., H.F. and the author of the Enzian classification J.K.), all with specific expertise and experience in diagnosing and treating endometriosis. Their inputs were included in a revised draft sent to all authors. After revision and discussion, a consensus was reached among the authors.

## Results

Six articles describing SR of MRI for endometriosis were identified, of which one approached the #Enzian classification (Table 1) [7, 20–24]. Five of the SR are itemized templates with key features. Five out of 6 SR organized pertinent pelvic structures into compartments (anterior, middle, posterior, others). Although not true anatomic “spaces,” the compartments mirror the gynecologist approach to surgical planning and may serve to organize a logical search pattern for the radiologist [22].

**Table 1:** Published MRI structured reports for endometriosis

Authors	Ref.	Template report authors	Template report content	Template report structure
Jaramillo-Cardoso et al.	[21]	Radiologists, gynecologic surgeons	Provides a brief description of imaging features	MRI template based on 5 compartments (anterior, middle, posterior, adnexal, others)
Mattos et al.	[7]	Radiologists, clinicians, surgeons	Provides a detailed description of imaging features	US & MRI template based on compartments (anterior, middle and posterior) and intestinal sites of endometriosis
Manganaro et al.	[20]	Radiologists	Adds some changes to #Enzian	MRI SR based on #Enzian
Feldman et al.	[24]	Multidisciplinary endometriosis team	Provides a detailed description of imaging features	MRI template based on 3 compartments (anterior, middle, posterior) and additional sites of endometriosis
Barbisan et al.	[23]	Radiologists, clinicians, surgeons	Provides a detailed description of imaging features	MRI template based on 3 compartments (anterior, middle, posterior)
Sud et al.	[22]	Radiologists	Provides a detailed description of imaging features	MRI template based on 3 compartments (anterior, middle, posterior) and additional sites of endometriosis

The comprehensiveness of the information provided was variable among previously published SR; for instance, adenomyosis was specifically mentioned only in two SR, while round ligaments were mentioned in only one of the SR. There was some heterogeneity regarding the nomenclature of anatomical spaces and other locations where endometriosis can be detected.

Manganaro et al. [20] SR, followed the #Enzian classification layout, suggesting a few pertinent changes to the #Enzian(m) in order to reach a more suitable evaluation for MRI, namely regarding tubal evaluation. The authors proposed the inclusion of the following parameters: presence or absence of sactosalpinx, the largest diameter measured in the point of the greatest distension of the tube and specify the content of the sactosalpinx (hematic, simple fluid or corpuscular).

### Proposal of a SR for MRI of endometriosis incorporating the #Enzian classification: rationale

According to the #Enzian classification aims, endometriosis can be mapped with one single classification system, applicable by non-invasive (US, MRI) and invasive methods, thereby enabling the use of one common language for describing endometriosis [14]. Although this unifying concept is very attractive, being the #Enzian classification primarily a surgical classification system, it needs to be adapted to the specificities of US and MRI.

It is important to clarify, that #Enzian is a classification system for endometriosis and even #Enzian based on MRI (#Enzian (m)) do not intend and cannot replace the MRI report itself, as the goals and content of these two perspectives of endometriosis mapping are distinct, being characterized by different levels of detail. On the other hand, integrating the #Enzian classification in the MRI report adds value to the report, the core product of diagnostic radiology and can improve the transmission of information.

### Applicability of #Enzian classification to the MRI

**Fig. 1:**
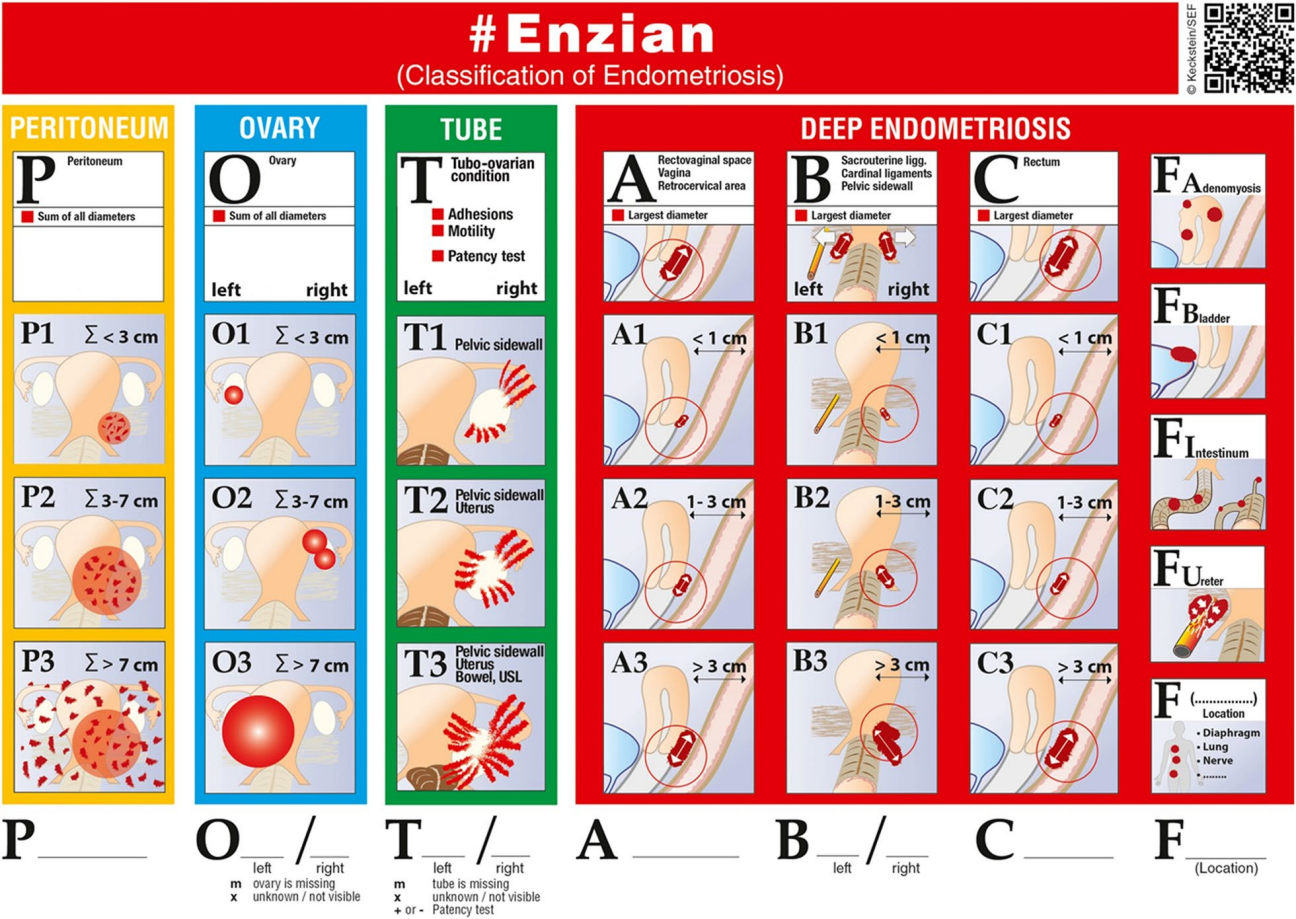
The #Enzian classification of endometriosis—an overview with potentially affected locations and disease extension (reproduced with permission from Keckstein et al. [14])

To delineate the SR, the compatibility between the #Enzian classification and MRI evaluation needs to be addressed, while revisiting the classification (Fig. 1).

### Peritoneum (P)

MRI has poor results in demonstrating peritoneal endometriosis, presenting sensitivity, specificity, accuracy, and PPV of 14%, 76%, 70% and 7%, respectively, in a recent study [25], in line with previous literature [26]. Peritoneal endometriotic implants can be visualized when present in the uterine wall and/ or on ovaries surface.

While MRI has a low diagnostic sensibility but a high specificity for the diagnosis of endometriotic peritoneal implants, pre-operative diagnosis of compartment P does not usually change the surgical strategy and therefore small undefined peritoneal lesions could be neglected [25].

*Applicability of #Enzian classification to the MRI: partial.*

### Ovary (O)

According to the #Enzian classification, ovarian endometriosis includes all the endometriomas and infiltrating ovarian surface foci (> 5 mm). The division into different stages of ovarian endometriosis according to the sum of endometrioma(s) size (Σ < 3 cm, Σ 3–7 cm, Σ > 7 cm) is based on the treatment approach. Endometriomas smaller than 3 cm are usually not treated surgically, whereas lesions larger than 3 cm are more often subjected to surgery by stripping or vaporization procedures. A diameter larger than 7 cm is recommended as the critical limit of size for organ-preserving surgery [14]. The sum of the endometriomas size is a simple quick statement about the volume of disease in each ovary.

*Applicability of #Enzian classification to the MRI: yes.*

### Tubo-ovarian condition (T)

#### Adhesions

Adhesions can be induced by endometriosis as a result of the inflammatory process of the disease [27]. The #Enzian classification includes the adhesions that can affect the mobility of ovaries and tubes. These adhesions are divided into three categories, according to their multiple locations (tubo-ovarian, pelvic side wall, uterus, uterosacral ligaments (USL) and bowel) and possible combinations, being cumbersome for MRI evaluation and reporting:

- T1: adhesions between the ovary and pelvic side wall ± tubo-ovarian adhesions.
- T2: T1 + adhesions between the ovary and the uterus. In alternative, isolated adhesions between the adnexa and uterus.
- T3: T2 + adhesions to the USL and/or bowel. In alternative, isolated adhesions between the adnexa and the USL and/or bowel.

On MRI, adhesions are seen as spiculated low signal intensity strands of variable thickness extending between the organs on both T1W and T2W images. Adhesions can also be identified by indirect signs, such as distortion of normal anatomy, including elevation of the posterior vaginal fornix, posterior and lateral displacement of the uterus, ovaries or both, loss of fat planes between the structures without a clear interface, hydrosalpinx, angulation of bowel loops, transition points in bowel diameter, and loculated fluid collections [11].

Adhesions may be too thin to be visualized on MRI [28]. Kataoka et al. [29] reported a mean sensitivity of 77.8%, a mean specificity of 50.0%, and a mean accuracy of 76.3% for the detection of adhesions. In particular, in the evaluation of peri-ovarian adhesions, MRI showed low sensitivity (right ovary 47.8%; left ovary 59.2%) and a specificity, respectively, of 88.1% and 85.2%.

Of note, adhesions can appear in other pelvic locations, namely in the vesico-uterine pouch, not specifically mentioned in the #Enzian classification.

*Applicability of #Enzian classification to the MRI: partial.*

#### Mobility of the ovaries and fallopian tubes

MRI is not able to assess the mobility of the tubo-ovarian unit [20]. This represents a major difference compared to transvaginal ultrasound (TVUS) and a limitation of MRI evaluation.

*Applicability of #Enzian classification to the MRI: no.*

#### Tubal patency

According to the #Enzian classification, the evaluation of tubal patency is optional and may be documented with hysterosalpingo contrast sonography and/or perturbation during surgery [14].

MRI-hysterosalpingography (MR-HSG) has been recently proposed as an innovative technique to investigate tubal patency and intracavitary anomalies. Different studies have been published using 1.5 T and 3.0 T magnets demonstrating the feasibility of MR-HSG and reporting high sensitivity, specificity and accuracy [30, 31]. However, this was not widely accepted and is rarely used in the clinical practice for two primary reasons: a lengthening of the examination and the impossibility to carry out an examination in real time.

*Applicability of #Enzian classification to the MRI: potential.*

### Deep endometriosis

The description of DIE follows a three axes organization: compartment A refers to a craniocaudal axis, compartment B refers to a mediolateral axis, and compartment C refers to antero-posterior axis (mainly rectal infiltration).

#### Compartment A: rectovaginal space, vagina, retrocervical area

Compartment A assesses the involvement of the retrocervical area, the posterior vaginal fornix and the rectovaginal space. The maximal diameter of the lesion is measured in the sagittal plane, and it is classified as follows: A1 =  < 1 cm, A2 = 1–3 cm, A3 =  > 3 cm. In case of multiple involvement of these structures, the maximum diameter of the whole involvement should be measured [14]. On MRI, the A compartment of DIE can be evaluated and measured on the sagittal T2W images [25, 26].

*Applicability of #Enzian classification to the MRI: yes.*

#### Compartment B: uterosacral ligaments, cardinal ligaments, pelvic side wall

Compartment B assesses the medio-lateral axis, mainly including the parametrial area and the uterosacral ligaments. The description of the lesions in this compartment is classified as follows: B1 =  < 1 cm maximum diameter, B2 = 1–3 cm, B3 =  > 3 cm. Although the involvement of the B compartment may cause hydronephrosis, the ureteral involvement and hydronephrosis are classified as FU compartment [14]. On MRI, the B compartment can be evaluated and measured on the axial T2W images, possibly with small field of view [25, 26].

*Applicability of #Enzian classification to the MRI: yes.*

#### Compartment C-rectum

Compartment C assesses the presence and extension of lesions in the anterior wall of the rectum (up to 16 cm from the anal verge). The maximal diameter of the lesion is measured in the sagittal plane, along the axis of the rectum, and it is classified as follows: C1 =  < 1 cm maximal diameter, C2 = 1–3 cm, C3 =  > 3 cm. In cases of multifocal lesions, the sum of the total length involved should be measured [14]. On MRI, the C compartment can be evaluated and measured on the sagittal T2W images [25, 26].

*Applicability of #Enzian classification to the MRI: yes.*

### Adenomyosis and other extragenital deep endometriosis

#### Adenomyosis (FA)

There is a high incidence of adenomyosis in patients with DIE, concurring to the infertility problems of the patients that undergo surgical treatment for DIE. For this reason, evaluation of adenomyosis is included in the #Enzian classification.

MRI has high diagnostic accuracy in the detection of adenomyosis, showing a high sensitivity (77%) and specificity (89%) [32]. Internal adenomyosis is characterized by ectopic endometrial glands and stroma displaced in the internal myometrium, resulting in hypertrophy and hyperplasia of the adjacent smooth muscle cells [33]. External adenomyosis is defined as a nodule/plaque that infiltrates into the myometrium from the serosal surface in continuity with DIE [11].

*Applicability of #Enzian classification to the MRI: yes.*

#### Bladder (FB)

Bladder DIE is defined by the presence of endometriotic tissue invading the detrusor muscle of the bladder, sometimes protruding into the lumen, with invasion of the mucosal layer. Lesions on the peritoneal surface of the bladder are considered peritoneal endometriosis [27]. MRI has high accuracy in detection of bladder endometriosis [34].

*Applicability of #Enzian classification to the MRI: yes.*

#### Intestinum (FI)

The #Enzian classification describes DIE involving other bowel structures besides the rectum: lesions cranial to the rectosigmoid junction (above 16 cm from the annal verge) – sigmoid, transverse colon, cecum, appendix and small bowel [14]. Magnetic resonance enterography is useful in the evaluation of intestinal endometriotic lesions and can be performed in addition to the conventional MRI protocol, when required [35].

*Applicability of #Enzian classification to the MRI: yes.*

#### Ureter (FU)

The #Enzian classification includes ureteral involvement by endometriosis. MRI has high accuracy in detection of ureteral endometriosis [36].

*Applicability of #Enzian classification to the MRI: yes.*

#### F (…)

According to the #Enzian classification, lesions on other locations, such as the abdominal wall, diaphragm, lung, and nerve, are annotated as F (…), directly in brackets [14].

MRI is useful in the detection and diagnostic work-up of abdominal wall endometriosis [37]. MRI is an excellent imaging modality with high accuracy for identification of diaphragmatic endometriotic implants [38], and a reliable imaging modality for detecting neural involvement of endometriosis [39]. Additionally, MRI is a good option for the characterization of pleural endometriotic implants and hemorrhagic pleural effusion [40]. Regarding evaluation of lung parenchymal endometriotic nodules, a very rare manifestation of endometriosis [40], MRI has low resolution compared to computed tomography (CT) and CT would be more accurate in the evaluation of parenchymal disease.

*Applicability of #Enzian classification to the MRI:* yes, (with exception of lung parenchymal involvement).

### Proposal of a SR for MRI of endometriosis incorporating the #Enzian classification: content

The SR template (Additional file 1) should integrate the #Enzian classification criteria and at the same time have the level of detail needed in the characterization of the disease and provided by MRI.

A conventional structured layout with standard headers is recommended: *title of examination, clinical details/indication*, *technique, comparison*, *imaging findings* and c*onclusion*, following the guidelines of the European Society of Radiology for radiological reporting [41].

The section on imaging findings was divided in sub-sections, according to the disease imaging features and relevant pre-operative information to include. A complete description of the lesions and measurements (in two or three orthogonal planes) should be performed. For instance, although round ligaments are not specifically mentioned in the #Enzian classification, involvement by endometriosis should be reported. Round ligaments have intraperitoneal and extraperitoneal components, both of which can be involved by DIE [42], being especially important to report extraperitoneal disease, as this is not routinely explored during surgery [23].

**Fig. 2:**
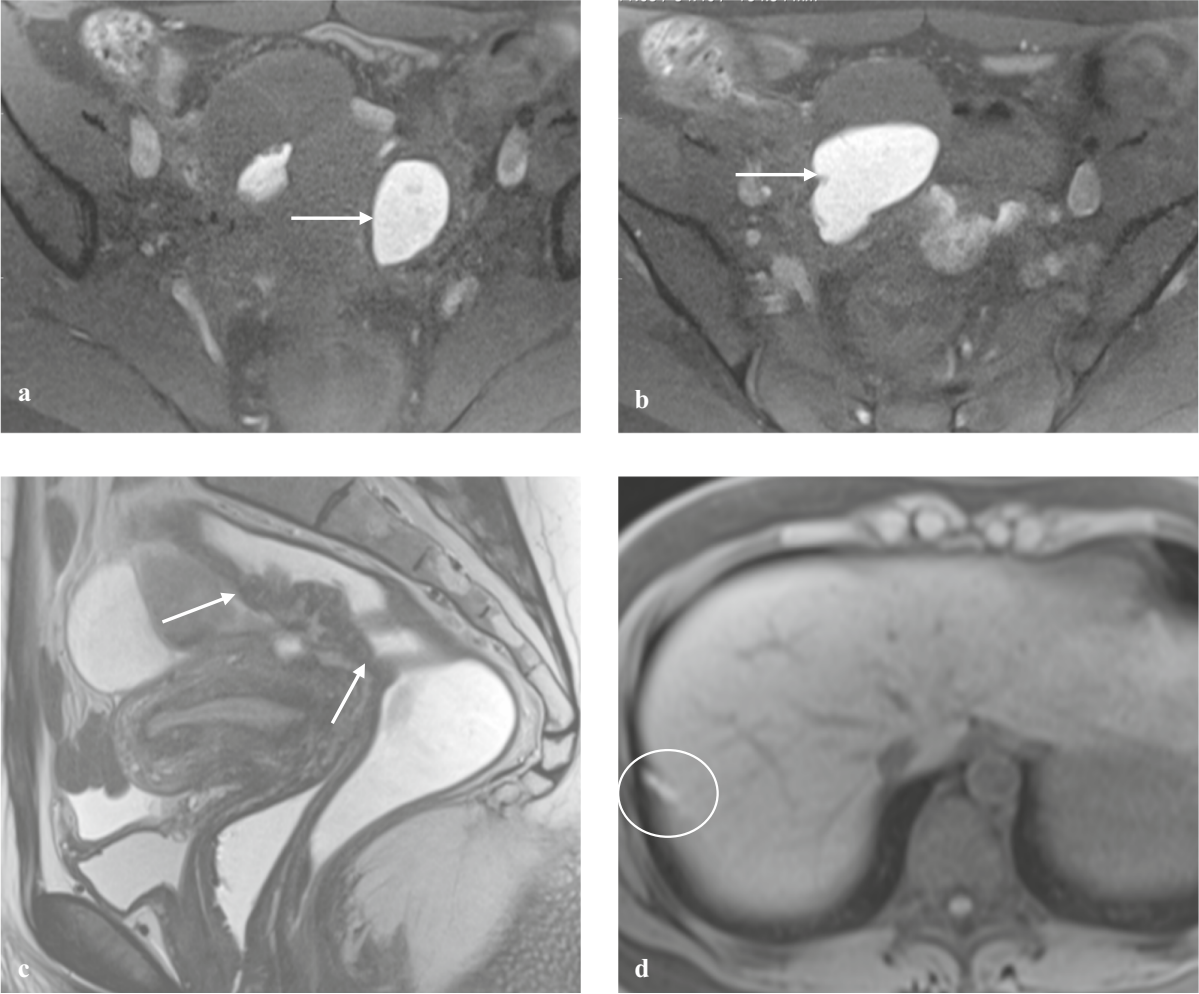
Example of the application of the #Enzian (m) classification, with MR images and coding. 33-year-old patient with pelvic and diaphragmatic endometriosis. **a**, **b** Axial fat-saturated T1W images demonstrate bilateral endometriomas, on the left ovary with 3.2 cm and on the right ovary with 5.1 cm of great axis (arrows). **c** Sagittal T2W image shows a 4.5 cm long, fan-shaped lesion of the upper rectum, in keeping with rectal endometriosis (between arrows). **d** Axial fat-saturated T1W image shows hyperintense spots (circle) in keeping with endometriosis lesions on the right hemidiaphragm. Disease classification according to #Enzian (m): O 2/2, C3, F(diaphragm)

**Fig. 3:**
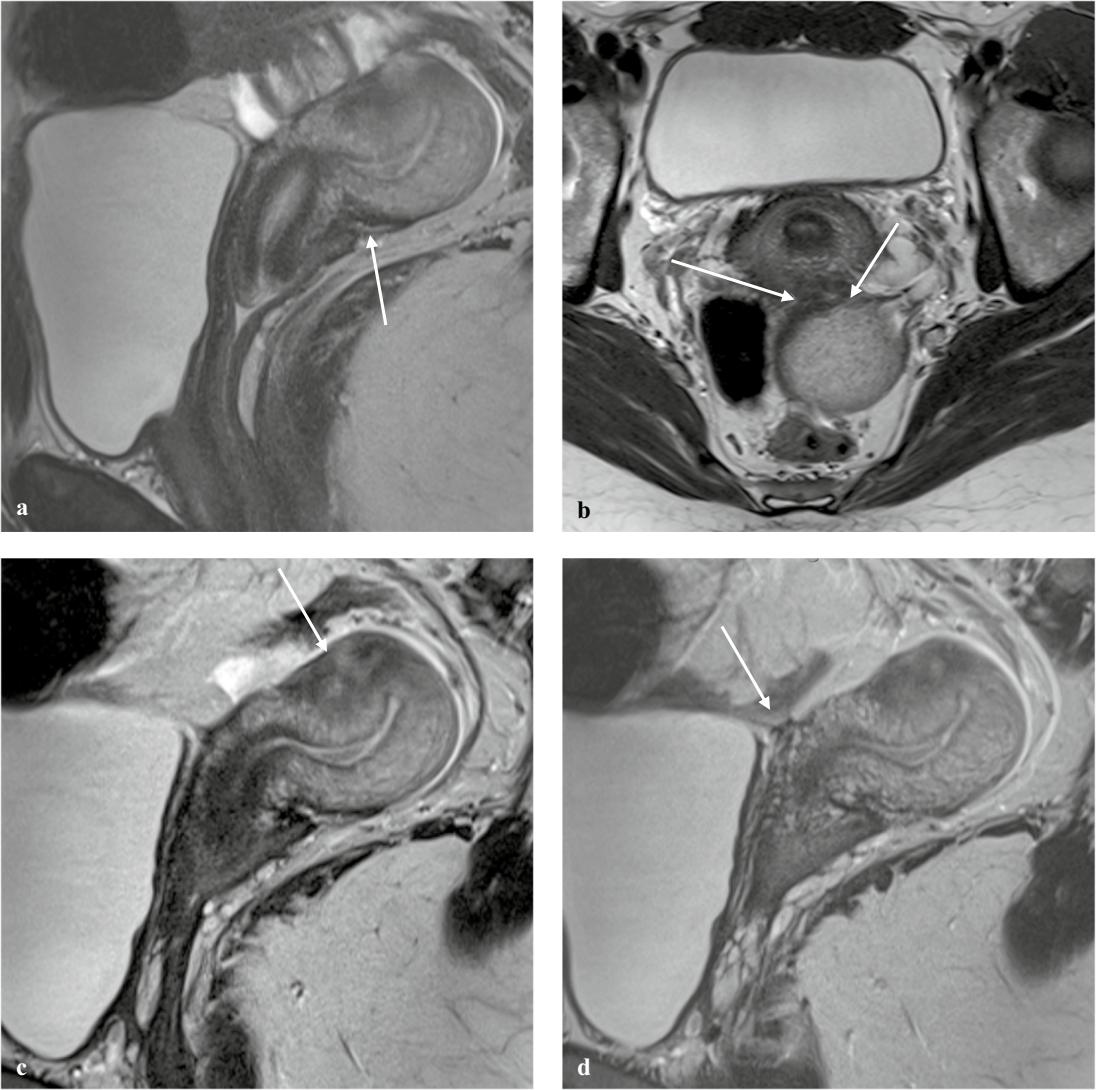
Example of the application of the #Enzian (m) classification, with MR images and coding. 22-year-old-patient with pelvic and small bowel endometriosis. **a** Sagittal T2W image shows a 0.5 cm endometriosis lesion in the rectovaginal space. **b** Axial T2W image demonstrates bilateral areas (approximately to 2 cm) of fibrotic thickening and low signal intensity in keeping with uterosacral ligamental involvement (arrows). **c** Sagittal T2W image shows focal adenomyosis on the anterior uterine wall (arrow). **d** Sagittal T2W image shows a focal thickening on the wall of a loop of ileum (arrow) in keeping with ileal endometriosis (arrow). Disease classification according to #Enzian (m): A1, B2/2, FA, FI

**Fig. 4:**
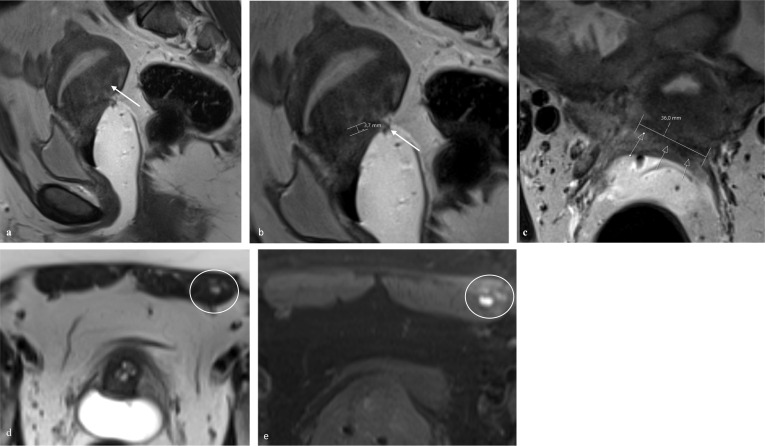
Example of the application of the #Enzian (m) classification, with MR images and coding. 38-year-old patient with abdominal wall endometriosis in the C-section scar and pelvic endometriosis. **a** Sagittal T2W image depicts an anteverted-retroflexed uterus. The posterior junctional zone of the uterus is thickened, in keeping with focal adenomyosis (arrow). **b** Axial T2W image demonstrate a plaque-like retrocervical endometriotic lesion, with low signal intensity, measuring about 3.6 cm in the axial plane (arrows). **c** Sagittal T2W image. The exact measurement of the retrocervical involvement on the sagittal plane, is challenging, due to the millimetric thickness of the lesion (about 0.4 cm). Elevation of the posterior vaginal fornix is seen (arrow). **d** Axial T2W image and **e** axial fat-saturated T1W image depict a nodular lesion (circles) in the left rectus abdominis muscle, showing high signal intensity foci on both sequences, suggestive of blood products. An US guided biopsy was performed and the histopathological examination confirmed an endometriotic nodule. Disease classification according to #Enzian (m): A1, FA, F (abdominal wall)

The MRI report should end with the #Enzian classification summarized in a code, as detailed in the classification. This code elegantly synthesizes the disease extension, in a standardized way, improving the clinical work, allowing for easy comparability between reports of the same patient or different patients and facilitating research. Examples of the application of the #Enzian (m) classification are provided (Figs. 2, 3, 4).

## Discussion

In the last years, several papers highlighted the increasing role of MRI in the diagnosis of endometriosis [43]. MRI shows high accuracy approaching criteria as triage and replacement test versus surgery, particularly for DIE [4].

The Enzian classification 2011 showed a good correlation between pre-operative MRI features and intraoperative findings in patients with DIE [44, 45]. Recent literature has also evaluated inter-reader agreement with this classification, showing varying results; Thomassin-Naggara et al.’s trial [16] consisted of 150 cases affected by DIE. They found excellent inter-reader agreement for A and C compartments but poor agreement for the B compartment. On the other hand, the trial conducted by Burla et al. [46], which consisted of 21 cases of DIE, showed an overall lower concordance, with a particularly weak inter-reader agreement for compartment C.

The #Enzian proposes a new comprehensive classification system, combining a complete staging of DIE with the evaluation of peritoneal/ovarian/tubal localizations and the presence of adenomyosis. Actually, the applicability of the #Enzian to MRI would benefit from some modifications that would render the classification more suitable for MRI evaluation. The most important limitation is the evaluation of tubo-ovarian condition. As reported in the classification, the subdivision of the adhesions in three different groups on the basis of the sites and of the involved organs remains complex and not reproducible. Additionally, MRI cannot assess the tubo-ovarian mobility and the tubal patency as defined in the classification [20]. According to a recent study of Manganaro et al. [20] including 60 patients, evaluating the applicability and reproducibility of the #Enzian classification to MRI, the concordance between readers (radiologists) regarding tubo-ovarian condition was poor and the worst of all compartments included in the #Enzian classification. Another limitation is the low sensibility of MRI in the diagnosis of peritoneal endometriosis [25]. Regarding reproducibility of the #Enzian classification applied to MRI, excellent inter-reader agreement for peritoneal implants, adnexal lesions and uterine adenomyosis was demonstrated and moderate concordance for DIE, highlighting that the correct assessment of DIE is related to reader’s expertise [20].

Despite the mentioned limitations, MRI allows a detailed evaluation of a complex and multifocal disease as endometriosis, covering in a single examination the majority of disease locations, including abdominal wall, ureteral, vesical and intestinal involvement, and even the thorax, if required. From this point of view, MRI is an exceptionally well-suited imaging modality to #Enzian classification, as this classification system incorporates virtually all the disease sites, thanks to the F (…) compartment.

Based on the authors’ experience, there are some additional practical considerations when applying the #Enzian classification to MRI reporting. First, accurate measurement of disease involvement of the USL can be challenging due to morphology which may present as asymmetrical (diffuse or focal) ligament thickening (unilateral or bilateral) or as a nodular lesion abutting the ligament [28]. The measurement in the case of thickening of USL has low inter-reader agreement, while in the presence of nodular lesion the measurement results easier. However, application of the #Enzian classification do not require an exact measurement. Instead, disease classification is based on broad size criteria, i.e., less than 1 cm (B1), 1–3 cm (B2) or more than 3 cm (B3), which makes reporting easier. Another area that radiologists may occasionally find troublesome is in trying to distinguish disease involvement between compartment A and C, in the presence of dense adhesions in posterior compartment resulting in cul de sac obliteration or when there is an initial infiltration of external layers of the rectum. Otherwise, in the cases presenting with a full thickness invasion of rectum wall, MRI can well differentiate the two compartments.

To the authors’ best knowledge, this is the first publication to propose an #Enzian classification-based MRI SR template for endometriosis, which allows unified coding of disease between surgeons and radiologists. This kind of organization is a novelty, as the SR we found in the literature followed the traditional organization in anterior, middle and posterior pelvic compartments, with some SR adding other components, such as adnexal compartment or additional sites of endometriosis.

Finally, there is a learning curve when adopting #Enzian classification into routine reporting practice, which may initially increase reporting time. However, the authors have found that through regular application of #Enzian to reporting and dialog between surgeons and radiologists, radiological interpretation can be refined. The use of a SR further simplifies this process.

## Conclusion

Aside from tubo-ovarian disease, much of the #Enzian classification is directly applicable to MRI reporting. A SR for MRI based on the #Enzian classification allows for a common shared language between radiologists and surgeons/gynecologists. The integration of the #Enzian classification in the MRI report itself can simplify the radiologist reporting work and improve the clinical work, allowing for easy comparability between reports of the same patient or different patients and facilitating research.

## Supplementary Information

**Additional file 1**: Structured MRI report template for endometriosis in correlation with the #Enzian classification.

## Data Availability

Not applicable.

## Abbreviations

DIE: Deep infiltrative endometriosis
ESUR: European Society of Urogenital Radiology
MRI: Magnetic resonance imaging
SR: Structured report
T1W: T1-weighted
T2W: T2-weighted
TVUS: Transvaginal ultrasound
US: Ultrasound
