# Supplementary material for: MRI of endometriosis in correlation with the #Enzian classification: applicability and structured report
Source: Insights Imaging. 2023 Jul 5;14:120. doi: 10.1186/s13244-023-01466-x (PMC10323073; doi:10.1186/s13244-023-01466-x)
Supplement: Supplementary file 1 — Additional file 1: Structured MRI report template for endometriosis in correlation with the #Enzian classification. [file 13244_2023_1466_MOESM1_ESM.pdf]

MRI of endometriosis in correlation with the #Enzian classification: applicability and structured report

ELECTRONIC SUPPLEMENTARY MATERIAL

Structured MRI report template for endometriosis in correlation with the #Enzian classification.

TITLE OF EXAMINATION

CLINICAL DETAILS/INDICATION

(Symptoms, relevant findings on gynecologic examination, medications, clinical suspicion/diagnosis, prior surgeries …)

TECHNIQUE

(MRI sequences, patient preparation, imaging quality issues)

COMPARISON

(With previous MRI examinations or correlation with previous US/CT examinations)

IMAGING FINDINGS

PERITONEUM (P)

Endometriotic peritoneal implants
Σ <3cm, Σ 3-7cm, Σ >7cm

OVARY (O)

Right: missing (m); unknown/not visible (x)
Left: missing (m); unknown/not visible (x)

Endometriomas and infiltrating ovarian surface foci
Right ovary: Σ <3cm, Σ 3-7cm, Σ >7cm
Left ovary: Σ <3cm, Σ 3-7cm, Σ >7cm

Additional information: location and size of ovaries, presence/absence of ovarian follicles, ovarian lesions besides endometriosis

TUBE (T)

Right: missing (m); unknown/not visible (x)
adnexal adhesions
Left: missing (m); unknown/not visible (x)
adnexal adhesions

Additional information: hematosalpinx, hydrosalpinx

COMPARTMENT A (craniocaudal axis)

- **RECTOVAGINAL SPACE**
- **VAGINA**
- **RETROCERVICAL AREA**

A1<1cm; A2 1-3cm; A3 >3cm

COMPARTMENT B (mediolateral axis)

- **USL**
- **PARAMETRIAL INVASION**
- **PELVIC WALL INVASION**

Right: A1<1cm; A2 1-3cm; A3 >3cm
Left: A1<1cm; A2 1-3cm; A3 >3cm

COMPARTMENT C (anteroposterior axis)

- **RECTUM**

C1: <1cm; C2: 1-3cm; C3: >3cm

Additional information: distance to anal verge, depth of invasion, % of circumferential extent, luminal narrow (yes/no), distance between lesions (if multiple)

F ADENOMYOSIS

Yes/no

Additional information
-junctional zone: thickness (mm) & characteristics; external/internal
-uterus size and position

F BLADDER

Yes/no

Additional information: detrusor muscle/urothelium invasion, location with respect to UVJ and trigone, vesico-uterine pouch obliterated/non-obliterated

F INTESTINUM

Yes/no

Possible locations: sigmoid, transverse colon, cecum, appendix and small bowel

F URETER

Yes/no

Additional information: length of involvement, distance to UVJ, hydroureter, hydronephrosis

F (…)

Possible locations: abdominal wall, cesarean scar, diaphragm, liver, lung, nerves, round ligaments, …

ADDITIONAL INFORMATION

Uterus/cervix/vagina/vulva/urethra findings; free fluid; other adhesions; other relevant findings, …

CLASSIFICATION #ENZIAN (m)

P…, O…, T..., A..., B..., C... F... F(...)

Keckstein J, et al. The #Enzian classification: A comprehensive non-invasive and surgical description system for endometriosis. Acta ObstetGynecol Scand. 2021; https://doi.org/10111/aogs.14099

CONCLUSION

Summary of the relevant findings. Orientations regarding the need of additional radiologic examinations/procedures.

The report content combines information organized according to the #Enzian classification, in bold, with additional information relevant for the completeness of the report.
Lesions site description and measurements in three orthogonal planes, whenever feasible, should be reported.
UVJ- ureterovesical junction.
